# Is a variant of uncertain significance always ‘insignificant’? A systematic review on PRF1 A91V in Hemophagocytic Lymphohistocytosis and comparative analysis with Still’s disease

**DOI:** 10.1186/s13023-026-04296-4

**Published:** 2026-03-07

**Authors:** İbrahim Yahya Çakır, Abdulsamet Erden, Ertuğrul Çağrı Bölek, M. Fatih Mulayim, Rahime Duran, İbrahim Karaduman, Esma Eseroğlu, Burak Karakaya, Büşra Köksoy, Ali Babazade, Gulsum Kayhan, Hamit Küçük, Mehmet Ali Ergun, Abdurrahman Tufan, Mehmet Akif Öztürk

**Affiliations:** 1 Department of Internal Medicine, Subdivision of Rheumatology, Gazi University, Ankara, 06560 Türkiye; 2 Department of Medical Genetics, Faculty of Medicine, Gazi University, Ankara, 06560 Türkiye

**Keywords:** Hemophagocytic lymphohistiocytosis, PRF1, Perforin, Still’s disease, Macrophage activation syndrome, Hyperferritinemia, Cytopenia

## Abstract

**Background:**

Hemophagocytic lymphohistiocytosis (HLH) is a life-threatening hyperinflammatory disorder that may arise secondary to rheumatic diseases such as Still’s disease, where macrophage activation syndrome (MAS) represents its clinical counterpart. The pathogenic significance of the PRF1 A91V variant remains uncertain, although functional data suggest partial perforin dysfunction and a possible contribution to late-onset or atypical HLH. This study aimed to clarify the clinical implications of the PRF1 A91V variant through a systematic review of published HLH and MAS cases and a comparative analysis with a single-center Still’s disease cohort.

**Results:**

A total of 20 studies, including 38 individual HLH or MAS cases carrying the PRF1 A91V variant, were identified. The median age at diagnosis was 22 years, and 18.4% of patients were homozygous. Fever (82.6%), splenomegaly (57.9%), and hepatomegaly (36.8%) were the most frequent clinical findings. Anemia (57.1%) and thrombocytopenia (85.7%) were the predominant laboratory abnormalities, accompanied by marked hyperferritinemia (median 9319 ng/mL). Compared with 43 active Still’s disease cases, PRF1-mutated HLH patients showed significantly higher rates of cytopenias, hepatomegaly, and central nervous system involvement, together with substantially elevated ferritin levels (9,193 vs 800 ng/mL, *p* = 0.0023), whereas C-reactive protein levels were comparable. Receiver-operating characteristic analysis identified a ferritin cutoff of 7000 ng/mL (sensitivity 63.2%, specificity 84.6%) as the optimal discriminator for PRF1 A91V positivity. In multivariate regression, ferritin ≥ 7,000 ng/mL remained the only independent predictor (OR 17.3, 95% CI 2.0–146.3, *p* = 0.009).

**Conclusions:**

Patients carrying the PRF1 A91V variant represent a distinct subgroup within the spectrum of hyperinflammatory syndromes. Extreme hyperferritinemia combined with cytopenias should raise suspicion for perforin-related HLH rather than cytokine-driven MAS or classic Still’s disease. Recognition of this variant as a risk-modifying allele may guide early genetic testing and therapeutic decisions, including consideration of advanced interventions in selected cases.

**Supplementary information:**

The online version contains supplementary material available at 10.1186/s13023-026-04296-4.

## Background

Hemophagocytic lymphohistiocytosis (HLH) is a multisystemic, life-threatening disorder characterized by a severe inflammatory response driven by increased phagocytic activity [1]. The pathophysiology of HLH involves uncontrolled immune activation, including hyperactivation of natural killer (NK) cells, cytotoxic T lymphocytes, and macrophages, leading to multi-organ damage. Although immunosuppressive therapies are the mainstay of treatment, other approaches such as intravenous immunoglobulin (IVIG), plasma exchange, and hematopoietic stem cell transplantation (HSCT), particularly in genetic cases, may be required for refractory patients [2].

HLH is classified into two main types based on etiology. Primary (familial) HLH results from genetic variants, whereas secondary HLH develops in the context of immune-mediated inflammatory disorders, malignancies, or infections [3]. Primary HLH typically presents at a younger age, while secondary HLH is more often seen in older patients with an identifiable underlying cause [3]. A distinct subtype of secondary HLH that arises in the setting of rheumatic diseases (primarily Still’s Disease) is known as macrophage activation syndrome (MAS) [4, 5].

Among known mutations that cause primary HLH, those involving the perforin gene (*PRF1*) are the most frequently identified. Mutations in other genes associated with cytotoxic T-cell-mediated killing mechanisms, such as *STX11*, *STXBP2*, and *UNC13D*, are also commonly reported [6, 7]. Perforin normally functions by mediating the permeability of cytotoxic granules of CD8+ T cells to kill target cells. In cases with *PRF1* variants, this mechanism is disrupted, leading to impaired target cell lysis and failure to terminate the immune response, thereby contributing to the hyperinflammatory state [8].

Although the clinical significance of the *PRF1* A91V variant remains uncertain, reports suggest that it may contribute to the pathogenesis of late-onset, relatively mild forms of disease [7]. Clinically, this variant has been linked to hyperinflammatory conditions, especially MAS [9]. While it is seen in some patients with Still’s disease, it has also been described, albeit less frequently, in other forms of primary HLH [9]. To better delineate the clinical significance and spectrum of this variant, we present a case from our clinic together with a systematic review–based cohort of patients with HLH or MAS carrying the A91V variant, and we compare their features with an idiopathic Still’s disease cohort followed at our institution.

## Methods

### Case presentation and literature review

**Fig. 1:**
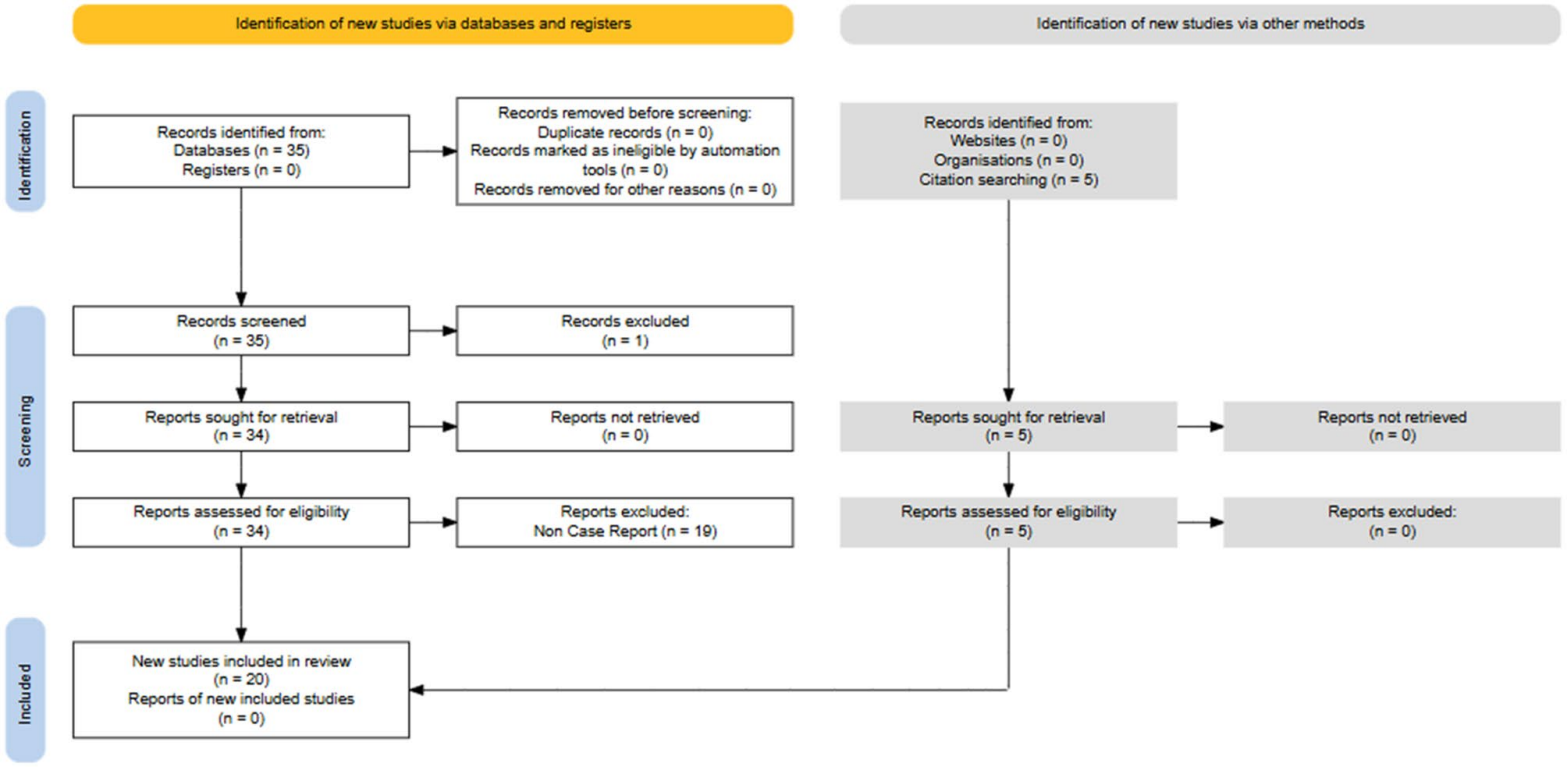
PRISMA 2020 flow diagram illustrating the study selection process. A total of 35 records were identified through database searching and an additional 5 studies were retrieved by citation screening, resulting in 20 eligible studies comprising 38 individual cases included in the final review

Building on the case observed in our clinic (described in the Supplementary File), we conducted a systematic literature review (SLR) to identify cases of HLH or MAS carrying the *PRF1 A91V* variant. We conducted a case-based review of the PubMed database between January 1, 1995, and February 1, 2025. The following search strategy was applied: ((A91V) OR (Ala91Val) OR (c0.272C > T)) AND ((Macrophage Activation Syndrome) OR (Hemophago*) OR (HLH) OR (MAS)). Only publications written in English were considered. To be eligible for inclusion, we selected original articles, case series, case reports, and letters to the editor that provided individual patient data describing cases with the *PRF1 A91V* mutation. Articles that did not contain patient-specific information, involved non-human studies such as cell cultures or animal models, or were published in languages other than English were excluded. The initial screening was based on the titles and abstracts of all retrieved records, followed by a full-text review of potentially relevant studies. In addition to database searches, the reference lists of all included full-text articles were manually reviewed to capture any additional eligible studies or case reports not retrieved through the initial search. This systematic review follows the Preferred Reporting Items for Systematic Reviews and Meta-Analyses (PRISMA) recommendations (Fig. 1) [10]. I.Y.C. and A.E. have completed the systematic literature review on August 28, 2025.

In addition, demographic characteristics, information regarding the mutation and its zygosity, clinical features, and treatment data of the cases were extracted. Anemia was defined as hemoglobin < 10 g/dL, leukocytosis as white blood cell count > 10,000/µL, leukopenia as white blood cell count < 4,000/µL, and thrombocytopenia as platelet count < 150,000/µL. These definitions were applied consistently across both the case reports included in the review and the Still’s disease cohort.

### Comparative analysis with single-center Still’s disease cohort

The Still’s disease cohort was created from our institutional medical records system in the Division of Rheumatology, Department of Internal Medicine, Faculty of Medicine, Gazi University, Ankara, Turkey. All patients aged 18 years or older who were diagnosed after January 1, 2014, were included. The patients were classified according to either the Yamaguchi or the Fautrel criteria [11, 12]. Baseline clinical and laboratory findings were recorded for all patients, and values were obtained at the time of diagnosis, when the disease was active. Disease activity was defined as the presence of at least one clinical domain suggesting activity, supported by an elevation in at least one acute-phase reactant. Comparisons were made between A91V HLH/MAS cases and active Still’s disease cases, as well as between A91V HLH cases and Still’s disease with MAS.

### Statistical analysis

Continuous and categorical variables were analyzed using standard methods. The distribution of continuous variables was assessed with the Kolmogorov–Smirnov test. Normally distributed variables were compared using the independent-samples t-test, while non-normally distributed variables were analyzed using the Mann–Whitney U test. Categorical variables were summarized as frequencies and percentages, and group comparisons were performed using the chi-square or Fisher’s exact test where appropriate. To evaluate the impact of independent variables on outcomes, binomial logistic regression analyses were performed. Variables with high collinearity were not included in the same regression model due to the limited sample size and quasi-complete separation observed among some predictors. Variables with a univariate *p*-value ≤ 0.20 and those considered clinically relevant in the comparison between HLH and Still’s disease groups were included in the logistic regression model.

Diagnostic cut-off values were determined by receiver operating characteristic (ROC) curve analysis. Disease status (A91V positivity) was used as the binary outcome variable, and ferritin concentration was entered as the test variable. The area under the curve (AUC) was calculated to assess overall discriminatory performance. The optimal threshold was defined as the value corresponding to 80% specificity and rounded to the nearest whole number for clinical applicability. In addition, sensitivity, specificity, positive predictive value (PPV), and negative predictive value (NPV) were calculated for selected thresholds.

A two-tailed *p*-value < 0.05 was considered statistically significant. All analyses were conducted using SPSS software (version 25; IBM Corp., Armonk, NY).

### Ethical considerations

The study was conducted in accordance with the principles of the Declaration of Helsinki [13]. Ethical approval for the use of the Still’s disease cohort was obtained from the Ankara University Faculty of Medicine Human Research Ethics Committee on October 25, 2024, under the application number 2024000624–1 (2024/624). Written informed consent was obtained from the patient for publication of the presented case.

## Results

A 20-year-old male initially diagnosed with Still’s disease experienced multiple relapses and subsequently developed macrophage activation syndrome (MAS) despite treatment with corticosteroids, methotrexate, and biologic agents, including anakinra and tocilizumab, which were also administered during MAS management. The diagnosis of MAS was established according to the 2016 EULAR/ACR/PRINTO classification criteria proposed by Ravelli et al., based on the presence of persistent fever, marked hyperferritinemia (ferritin > 684 ng/mL), elevated aspartate aminotransferase (AST > 48 U/L), and hypertriglyceridemia (>156 mg/dL) [14, 15]. Whole-exome sequencing revealed a homozygous PRF1 c0.272C > T (p.Ala91Val) variant. Partial disease control was achieved under anakinra maintenance therapy. The detailed clinical course of this patient is provided in the Supplementary Material.

A systematic search identified 35 articles, of which 34 were full-text available and were assessed for eligibility. Nineteen studies were excluded because they did not include case reports, while five additional studies were retrieved through citation searching. In total, 20 articles comprising 38 individual HLH or MAS cases with the *PRF1* A91V variant were included in the final analysis (Fig. 1). Key characteristics of the included studies and individual cases are summarized in Supplementary Table 1.

Among these 38 cases, half were male, with a median age at diagnosis of 22 years (IQR 18–45). The majority were heterozygous carriers, while 18.4% were homozygous. No other gene mutations besides PRF1 (including A91V and other variants) were reported. Fever (82.6%), splenomegaly (57.9%), and hepatomegaly (36.8%) were the most common clinical findings, followed by lymphadenopathy (27.8%) and Central Nervous System (CNS) involvement (27.0%). Laboratory abnormalities included anemia (57.1%) and thrombocytopenia (25.7%), with highly elevated inflammatory markers (median C-reactive protein [CRP] 57.8 mg/L; ferritin 9319 ng/mL). Corticosteroids were the most frequently used therapy (70.6%), followed by the HLH-2004 protocol (38.9%) and cyclosporine (23.5%). Overall, 17.6% of patients had previous episodes, 88.2% showed treatment response, yet 36.3% died during follow-up. Malignancy was identified in five patients, and infectious triggers were documented in 64.3% of those with available data (Table 1).

**Table 1:** Summary of demographic, clinical, laboratory, and therapeutic features of reported HLH cases with *PRF1* A91V mutation

Parameters*	
Female, n (%)	16/32 (50.0)
Age at Diagnosis median (25-75p)	22 (18–45)
A91V Homozygous, n (%)	7/38 (18.4)
Other *PRF1* Mutations, n (%)	10/38 (26.3)
Malignancy, n (%)	5/32 (15.6)
Infection, n (%)	9/14 (64.3)
**Clinical Findings**	
Fever, n (%)	19/23 (82.6)
Hepatomegaly, n (%)	7/19 (36.8)
Splenomegaly, n (%)	11/19 (57.9)
Rash, n (%)	6/18 (33.3)
Arthritis	0/5 (0)
Lympadenopathy, n (%)	5/18 (27.8)
Serosal Effusion, n (%)	5/20 (25.0)
Dyspnea, n (%)	4/20 (20.0)
Peripheral Edema, n (%)	2/17 (11.8)
Splenic Infarct, n (%)	1/19 (5.3)
Cardiovascular Failure, n (%)	3/20 (15.0)
Renal failure, n (%)	3/20 (15.0)
CNS Presentation, n (%)	10/37 (27.0)
**Laboratory Findings**	
Anemia, n (%)	12/21 (57.1)
Leucopenia, n (%)	8/21 (38.1)
Thrombocytopenia, n (%)	18/21 (85.7)
CRP (mg/L) (median, 25p − 75p)	57.8 (30.2–231.1)**
Ferritin (ng/mL) (median, 25p − 75p)	9319 (3008– 29,415)***
**Treatment**	
HLH-2004 protocole, n (%)	7/18 (38.9)
Glucocorticoid Pulse Therapy, n (%)	12/17 (70.6)
Cyclosporine (CsA), n (%)	4/17 (23.5)
Rituximab (RTX), n (%)	3/17 (17.6)
Intravenous Human Immunoglobulin G (IVIG), n (%)	2/17 (11.8)
IL1 Antagonists, n (%)	1/17 (5.9)
Other Chemotherapeutics, n (%)	2/17 (11.8)
Hematopoetic Stem Cell Transplantation, n (%)	4/19 (21)
Previous HLH Episode, n (%)	3/17 (17.6)
Treatment Response, n (%)	15/17 (88.2)
Death, n (%)	8/22 (36.3)

*The number of patients varied for each parameter due to non-standardized reporting in the case studies; analyses were based only on cases with available data

**CRP values were available for six patients

***Ferritin values were available for 18 patients

25 HLH patients with the A91V variant who were aged 18 years or older and had sufficient baseline demographic data were included for comparison with Still’s disease; together with our own case, 26 patients were comparatively analyzed with the first active episode of 43 patients with Still’s disease, and another five patients with Still’s disease in MAS. In comparison to active Still’s disease, HLH cases more frequently exhibited hepatomegaly (33.3% vs 2.8%; *p* = 0.021), CNS involvement (24.0% vs 0%; *p* = 0.0018), and hematologic abnormalities including anemia (72.7% vs 15.8%; *p* = 0.0007), leucopenia (54.5% vs 2.6%; *p* = 0.0002), and thrombocytopenia (81.8% vs 2.6%; *p* < 0.0001), along with markedly higher ferritin levels (9,193 ng/mL vs 800 ng/mL; *p* = 0.0023), while CRP levels (45.2 mg/L vs 96.0 mg/L; *p* = 0.884) were similar. In contrast, no statistically significant differences were observed between HLH and Still’s disease with MAS (Table 2)

**Table 2:** Clinical and laboratory comparison between PRF1-mutated HLH, active Still’s disease, and Still with MAS cases

Variables	Cases with PRF1 variant (n:26)*	Active Still Cases (n:43)	p₁ values	Still with MAS (n:6)	p₂ values
Age median (25-75p)	25 (19.3–47.5)	33 (24.5–44.0)	0.160	42 (30.3–53.8)	0.217
Female, n (%)	12/25 (48.0)	26/43 (60.5)	0.448	5/6 (83.3)	0.185
**Clinical Findings**					
Fever, n (%)	9/11 (81.8)	40/41 (97.6)	0.110	6/6 (100.0)	0.515
Hepatomegaly, n (%)	3/9 (33.3)	1/36 (2.8)	0.021	2/5 (40.0)	1.000
Splenomegaly, n (%)	5/9 (55.6)	10/36 (27.8)	0.135	4/5 (80.0)	0.580
Lymphadenopathy, n (%)	4/8 (50.0)	26/34 (76.5)	0.195	5/6 (83.3)	0.301
Rash, n (%)	4/8 (50.0)	35/41 (85.4)	0.044	3/5 (60.0)	1.000
Arthritis, n (%)	1/2 (50.0)	19/41 (46.3)	1.000	3/5 (60.0)	1.000
Serosal effusion, n (%)	4/10 (40.0)	7/40 (17.5)	0.197	5/6 (83.3)	0.145
CNS findings, n (%)	6/25 (24.0)	0/42 (0.0)	0,0018	0/6 (0.0)	0.309
**Laboratory Findings**					
Anemia, n (%)	8/11 (72.7)	6/38 (15.8)	0.0007	5/6 (83.3)	1.000
Leucopenia, n (%)	6/11 (54.5)	1/38 (2.6)	0.0002	3/6 (50.0)	1.000
Leucocytosis, n (%)	1/11 (9.1)	27/38 (71.1)	0.00035	2/6 (33.3)	0.515
Thrombocytopenia, n (%)	9/11 (81.8)	1/38 (2.6)	<0.0001	4/6 (66.7)	0.584
Thrombocytosis, n (%)	0/11 (0.0)	2/38 (5.3)	1.000	0/6 (0.0)	1.000
CRP median, (25p-75p) **	45.2 (23.7–122.6)	96.0 (22.8–126.5)	0.884	168.5 (105.8–300.3)	0.352
Ferritin median, (25p-75p)***	9193 (2069– 25,243)	800 (242–3214)	0.0023	10595 (2047– 33,936)	0.961

*Data for this group include both systematically identified cases from the literature and our own case

**CRP values were available for 4 HLH patients, 39 Active Still patients, and 6 Still MAS patients

***Ferritin values were available for 11 HLH patients, 39 Active Still patients, and 6 Still MAS patients

Note: p₁ values indicate comparisons between PRF1 A91V–associated HLH and active Still’s disease, while p₂ values represent comparisons between PRF1 A91V–associated HLH and Still’s disease with MAS

At a ferritin cutoff of 7000 ng/mL, the sensitivity and specificity for detecting PRF1 A91V positivity were 63.2% and 84.6%, respectively. This threshold was derived from ROC curve analysis performed indicating that ferritin levels ≥ 7000 ng/mL provided the best discrimination between A91V-positive and A91V-negative cases within the remaining cohort.

Regression analyses were performed to identify predictors of the A91V mutation in HLH. In univariate models, younger age (≤30 years) (OR 0.36, 95% CI 0.13–0.96, *p* = 0.043) and ferritin > 7000 ng/mL (OR 7.0, 95% CI 1.67–29.22, *p* = 0.008) were significantly associated with mutation positivity. In the multivariate model, a backward stepwise logistic regression (Likelihood Ratio method) was applied to identify independent predictors. Only ferritin > 7000 ng/mL remained a significant variable (OR 17.3, 95% CI 2.0–146.3, *p* = 0.009), indicating its strong association with mutation positivity. The detailed stepwise results are presented in Supplementary Table 2 (Table 3, Supplementary Table 2).

**Table 3:** Predictors of *PRF1* A91V mutation in HLH: regression analysis of diagnostic indicators

Parameters	Univariate Odds Ratio (OR)	p	Multivariate OR	p
Age ≥ 30 years	0.36 (0.13 - 096)	0.043		
Fever	0.09 (0.008 - 1.19)	0.069		
Splenomegaly	2.41 (0.55 - 10.42)	0.23		
Lympadenopathy	0.29 (0.06 - 1.39)	0.123	0.16 (0.02 - 1.32)	0.090
Rash	0.21 (0.04 - 1.02)	0.054	0.14 (0.01 - 1.13)	0.066
Serosal Effusion	1.88 (0.45 - 7.86)	0.38		
Ferritin ≥ 7.000	7.0 (1.67 - 29.22)	0.008	17.3 (2.0 - 146.3)	0.009

Note: Results represent the final model derived from backward stepwise logistic regression; detailed stepwise outputs are provided in Supplementary Table 2

## Discussion

Our analysis shows that individuals having this variant, especially those with adult-onset disease, commonly present with marked cytopenias, elevated ferritin levels, and systemic involvement such as hepatomegaly and neurological involvement. Although there is significant overlap in clinical and laboratory findings between Still’s disease, our results suggest that a combination of marked hyperferritinemia and profound cytopenias may help distinguish A91V-related HLH from both active Still’s disease and MAS secondary to Still’s disease. Ferritin levels equal to or above 7000 ng/mL were found to be an independent predictor of A91V-HLH, highlighting their value as a practical marker.

From a mechanistic perspective, Zhang et al. and earlier HLH analyses reaffirm that extreme ferritin elevations reflect macrophage and T-cell hyperactivation characteristic of cytotoxic dysfunction [16]. These findings suggest that not just the presence but the degree of ferritin elevation is clinically meaningful, potentially indicating impaired cytotoxic regulation related to perforin dysfunction. Particularly in younger patients with a Still-like presentation but extreme ferritin elevations and cytopenias, this should raise suspicion of HLH and early genetic testing for primary HLH genes. MAS, a life-threatening complication of Still’s disease, shares several clinical similarities with HLH. Macrophage activation syndrome (MAS), a life-threatening complication of Still’s disease, shares substantial clinical and laboratory overlap with hemophagocytic lymphohistiocytosis (HLH). Although PRF1 mutations are well-established causes of familial HLH, current genetic studies in Still’s disease and systemic juvenile idiopathic arthritis (sJIA) have not identified PRF1 as a primary disease susceptibility locus [17, 18]. Instead, these disorders are predominantly associated with variations in HLA class I and II regions and pathways such as CSF1/M-CSF, supporting an immunopathogenic model driven mainly by cytokine dysregulation and adaptive immune activation [17–19]. However, important insights emerge from pediatric-onset Still disease, which is considered part of the same disease spectrum as Still’s disease. Vastert et al. demonstrated that mutations in PRF1, including hypomorphic variants, were overrepresented in sJIA patients who developed MAS, suggesting that defects in cytotoxic pathways may lower the threshold for MAS rather than act as primary disease drivers [9]. Taken together, these findings support the concept that PRF1 variants function as permissive or risk-modifying factors within an inflammatory milieu, rather than deterministic causes of Still’s disease itself.

Even though PRF1-associated primary HLH, such as other monogenic forms, is typically diagnosed at a young age, most frequently during the first decade of life, it is a clinically distinctive and still under-recognized condition within the broader spectrum of hyperinflammatory syndromes in adulthood. Perforin is a pore-forming protein critical to immune defense, facilitating the delivery of granzymes from cytotoxic T cells and NK cells into target cells, where they induce apoptosis. Deficiency or dysfunction of perforin results in defective cytotoxic lymphocyte–mediated immune regulation, leading to persistent activation of macrophages and T cells, uncontrolled cytokine release, and a hyperinflammatory state, and represents the most common genetic cause of primary HLH [7, 8]. Recent comprehensive analyses have further highlighted the complexity of genotype–phenotype correlations in perforin-related disease. In a large registry and population-based study, Wegehaupt et al. demonstrated marked variability in disease penetrance among individuals carrying biallelic PRF1 variants involving A91V, including combinations with predicted loss-of-function alleles. Notably, many individuals with identical A91V/pLOF genotypes remained asymptomatic into late adulthood, while others developed severe and recurrent HLH, and neither perforin expression nor cytotoxicity assays reliably predicted clinical outcome. These findings support the concept of HLH as a threshold disease, in which residual cytotoxic capacity, environmental triggers, and inflammatory context collectively determine disease manifestation rather than genotype alone. In this framework, our findings do not suggest that PRF1 A91V is sufficient to cause disease, but rather that in the setting of pronounced hyperinflammation—such as extreme hyperferritinemia and cytopenias—this hypomorphic variant may act as a risk-modifying factor, contributing to phenotypic severity and HLH-like presentations in adults [20].

c0.272C > T (p.Ala91Val) variant in the *PRF1* gene, also homozygously detected in our patient, has been reported in population databases with an allele frequency of approximately 3.5% [19]. Even partial loss of perforin function, as seen in hypomorphic variants such as A91V, may impair immune regulation and contribute to hyperinflammatory states like HLH [8]. Functional data indicate that the A91V variant results in reduced perforin expression and protein stability, along with partial loss of cytotoxic function [7, 21–23].

This variant has been identified in patients with familial HLH, acute lymphoblastic leukemia, and also in healthy individuals, both in heterozygous and homozygous genotypes [24–26]. While its clinical impact remains controversial, including the possibility of a benign polymorphism, a variant of uncertain significance, or a disease-associated allele, accumulating evidence supports its role as a disease-modifying factor in some adult patients with HLH presentations [27].

According to our systematic review, most reported cases involved adult or elderly individuals, with HLH triggered by mild infections or coexisting rheumatologic diseases such as MAS [5, 27–34]. The observation that A91V is also found in asymptomatic carriers suggests incomplete penetrance, likely requiring additional genetic or environmental factors for clinical expression. The relatively late onset, partial response to immunosuppressive therapies, and absence of classic early-onset familial HLH features further support the view of A91V as a hypomorphic risk allele rather than a fully penetrant mutation.

Recent evidence further supports the diagnostic relevance of hyperferritinemia and cytopenias as key discriminators between cytokine-driven autoinflammation and hemophagocytic pathology. Bojan et al. (2021) emphasized in an extensive literature review that hyperferritinemia and thrombocytopenia are among the most consistent diagnostic features of macrophage activation syndrome (MAS) across rheumatologic contexts [35]. Similarly, Zhang et al. (2023) identified platelet count < 110 × 10^9^/L and ferritin > 548 ng/mL as independent early predictors of MAS in Kawasaki disease, underscoring the diagnostic value of these two parameters even in pediatric secondary HLH [36]. In Still’s disease, Ruscitti et al. (2023) showed that ferritin levels, alongside cytokines such as IFN-γ and IL-10, robustly discriminated MAS from uncomplicated AOSD, again highlighting ferritin’s role as a central biomarker of macrophage activation [37] Shiga et al. compared adult-onset Still’s disease, Still’s disease with MAS, and secondary HLH (mainly lymphoma-associated) and found that differentiating these entities can be challenging due to overlapping clinical and laboratory features. Ferritin levels were higher in Still’s disease, particularly in MAS cases, reaching median values around 35,000 ng/mL, although the difference versus secondary HLH did not reach statistical significance. The authors did not perform genetic screening, leaving open the possibility that some MAS patients could carry hypomorphic cytotoxic pathway variants such as PRF1 A91V [38]. In our study, ferritin ≥ 7,000 ng/mL was an independent predictor of A91V-HLH (OR 17.3, 95% CI 2.0–146.3, *p* = 0.009), suggesting that extreme hyperferritinemia, especially when accompanied by cytopenias, may indicate perforin-related HLH rather than cytokine-driven MAS or classic Still’s disease. In line with our findings, cytopenias were more frequent in the HLH group, whereas rash was more common among Still’s disease patients, with no significant difference in splenomegaly or lymphadenopathy between the two groups.

Our literature review provides valuable insights into the potential role of this rare variant in the clinical features of HLH and MAS. Furthermore, as also observed in our presented case referred to the rheumatology clinic, its comparative evaluation with Still’s disease and associated MAS presentations offers clinically relevant messages for physicians by highlighting important diagnostic distinctions and raising awareness. Nonetheless, several limitations should be considered. The HLH data were derived from published case reports and series, which lacked standardized clinical, laboratory, and genetic documentation, leading to heterogeneity and incomplete datasets. In this study design, it was not possible to evaluate other potentially unreported pathogenic genes or risk alleles beyond PRF1, as well as multigenic interactions and environmental factors. The retrospective design also introduces potential reporting and selection biases. Moreover, the Still’s disease and MAS patients in our cohort were not routinely genetically screened, so the possibility of undetected monogenic or polygenic predispositions cannot be excluded. Pathobiologic validation with functional tests for the A91V variant was unavailable in most cases, limiting conclusions about direct genotype–phenotype correlations. In addition, the number of patients with Still’s disease-related MAS was relatively small, which may have limited the ability to distinguish between *PRF1* HLH and MAS adequately. Similarly, the small number of HLH cases and statistical issues limited the ability to build a robust multivariate regression model. Despite these challenges, our findings offer critical clinical insights and establish a basis to test in multicenter prospective studies in the future.

In summary, *PRF1* A91V–associated HLH or MAS appears to form a distinct yet often overlooked subset within the spectrum of hyperinflammatory diseases. The A91V variant should therefore be considered as a risk-modifying or potentially pathogenic allele, not a benign polymorphism. Patients carrying this variant tend to present with profound cytopenias and marked hyperferritinemia, frequently without the typical features of Still’s disease such as rash, arthritis, or lymphadenopathy. In individuals who initially present with Still-like disease or MAS but show unusually high ferritin levels (≥7000 ng/mL) together with one or more cytopenias, an underlying perforin-related disorder should be suspected. For selected patients with recurrent or treatment-refractory disease, early identification may even allow consideration of advanced therapeutic options such as hematopoietic stem cell transplantation.

## Conclusion

This study demonstrates that the PRF1 A91V variant delineates a clinically relevant, yet often underrecognized, subgroup within the spectrum of hyperinflammatory disorders. Our findings do not support PRF1 A91V as a deterministic cause of disease; rather, consistent with recent population-based and registry data showing highly variable penetrance among A91V carriers, this variant appears to function as a risk-modifying allele whose clinical impact depends on inflammatory context and disease threshold. In this setting, patients presenting with Still-like disease who exhibit profound cytopenias and extreme hyperferritinemia represent a phenotype in which perforin-related immune dysregulation should be strongly considered. The identification of ferritin ≥ 7,000 ng/mL as an independent predictor of PRF1 A91V–associated HLH underscores ferritin’s value not only as a biomarker of macrophage activation but also as a clinical indicator of cytotoxic pathway involvement. Recognizing PRF1 A91V within a threshold-based HLH–MAS continuum refines current diagnostic reasoning and supports targeted genetic testing in patients with atypical, severe, or treatment-refractory presentations. While routine genetic screening or preemptive transplantation in asymptomatic carriers is not justified, early molecular evaluation in selected high-risk phenotypes may inform monitoring strategies and, in carefully selected cases, consideration of advanced therapies such as hematopoietic stem cell transplantation.

## Electronic supplementary material

Below is the link to the electronic supplementary material.

Supplementary Material 1

## Data Availability

All data generated or analyzed during this study are included in this published article and its supplementary information files. Additional details are available from the corresponding author upon reasonable request.

## Abbreviations

AUC: Area Under the Curve
CI: Confidence Interval
CNS: Central Nervous System
CRP: C-Reactive Protein
CsA: Cyclosporine A
DNA: Deoxyribonucleic Acid
EBV: Epstein-Barr Virus
FHLH: Familial Hemophagocytic Lymphohistiocytosis
HLH: Hemophagocytic Lymphohistiocytosis
HSCT: Hematopoietic Stem Cell Transplantation
IFN-γ: Interferon Gamma
IL-1: Interleukin-1
IL-6: Interleukin-6
IL-10: Interleukin-10
IQR: Interquartile Range
IVIG: Intravenous Immunoglobulin
MAS: Macrophage Activation Syndrome
NK: Natural Killer (cell)
NPV: Negative Predictive Value
OR: Odds Ratio
PRF1: Perforin-1 Gene
ROC: Receiver Operating Characteristic
SLR: Systematic Literature Review
SPSS: Statistical Package for the Social Sciences
sJIA: Systemic Juvenile Idiopathic Arthritis
