# Supplementary material for: Is a variant of uncertain significance always ‘insignificant’? A systematic review on PRF1 A91V in Hemophagocytic Lymphohistocytosis and comparative analysis with Still’s disease
Source: Orphanet J Rare Dis. 2026 Mar 7;21:150. doi: 10.1186/s13023-026-04296-4 (PMC13081438; doi:10.1186/s13023-026-04296-4)
Supplement: Supplementary file 1 — Supplementary Material 1 [file 13023_2026_4296_MOESM1_ESM.docx]

**Case Presentation**

A 20-year-old male patient first presented at the age of 18 with complaints of persistent high-grade fever for two months (maximum 39.7 °C), unintentional weight loss of 10 kg, pruritic pink skin rash, and diffuse arthralgia. Laboratory tests revealed an erythrocyte sedimentation rate (ESR) of 127 mm/hour, C-reactive protein (CRP) of 398 mg/L, and serum ferritin levels exceeding the laboratory’s upper limit of detection. Hemoglobin was measured at 7.4 g/dL, white blood cell (WBC) count at 12.7 ×10³/μL, and polymorphonuclear leukocyte (PMNL) count at 10.2 ×10³/μL. Liver enzymes were elevated to 6–7 times the normal range, while albumin was 3.4 g/dL.Triglycerides were 162 mg/dL, and fibrinogen was 694 mg/dL. Renal function and electrolytes were within normal limits.

Transthoracic echocardiography excluded infective endocarditis. Thoracoabdominal computed tomography (CT) demonstrated bilateral axillary, paraaortic, paracaval, mesenteric, and iliac–obturator lymphadenopathy, accompanied by hepatosplenomegaly, without any abscess formation or mass lesion suggestive of malignancy. A lymph node excision biopsy obtained from the right inguinal region revealed reactive lymphoid hyperplasia without neoplastic infiltration. Serological testing for Epstein–Barr virus (EBV), cytomegalovirus (CMV), parvovirus B19, human immunodeficiency virus (HIV), and hepatitis B and C was negative. Interferon-γ release assay (IGRA) and mycobacterial cultures were also negative, effectively excluding tuberculosis. Additional autoimmune and rheumatologic serologies, including ANA, anti-dsDNA, rheumatoid factor, and ANCA, were all negative.

The diagnosis of Still’s disease was established according to both the Yamaguchi and Fautrel classification criteria. [(1,2)](https://www.zotero.org/google-docs/?78sWQF)

Based on the overall clinical and laboratory findings, a working diagnosis of Still’s disease was established, and intravenous anakinra (3 × 100 mg/day for three days) was initiated, followed by methylprednisolone (1 mg/kg/day). The patient was counseled regarding the potential risks of incomplete therapy and the importance of follow-up. Corticosteroid tapering was recommended, but the patient elected to leave the hospital voluntarily before the diagnostic work-up and treatment plan were fully completed, and did not attend subsequent follow-up visits.

Four months later, he was re-admitted with fever, skin rash, and arthritis in the bilateral wrists. He had been using methylprednisolone irregularly at a high dose (32 mg/day). His HbA1c was 7.3%, and his blood glucose was >300 mg/dL. Lymph node biopsy was reported as reactive, excluding lymphoproliferative disorders and specific infections. After exclusion of lymphoproliferative and infectious diseases, the diagnosis of Still’s disease was confirmed. Oral methotrexate and metformin were initiated.

Another four months later, the patient presented for a third time, exhibiting cushingoid features, restricted movement in both wrists, fever (39.5 °C), widespread arthralgia, and purple striae on the skin. Laboratory findings included ferritin of 12,792 ng/mL, HbA1c of 12.1%, WBC of 17.7 ×10³/μL, PMNL of 13 ×10³/μL, and CRP of 163 mg/L. Subcutaneous methotrexate and insulin therapy were started.

Eight months later, he was admitted to the emergency department with confusion, diarrhea, and dysarthria. Brain MRI showed no acute pathology. Ferritin was 91,391 ng/mL, CRP was 291 mg/L, LDH was 1212 U/L, hemoglobin was 9 g/dL, platelet count was 39 ×10³/μL, and lymphocyte count was 0.8 ×10³/μL. Liver enzymes were elevated by 2–3 times, and albumin was 3 g/dL. The patient fulfilled MAS classification criteria according to the 2016 EULAR/ACR/PRINTO criteria (Ravelli et al.), and also met HLH-2004 criteria. He underwent cytokine adsorption therapy for three consecutive days and received high-dose intravenous methylprednisolone, followed by a five-day course of anakinra. A favorable clinical response was achieved, after which weekly tocilizumab was initiated. Whole-exome sequencing (WES) was subsequently performed for genetic evaluation.

Eight months later, the patient returned with blurred vision, arthralgia, nausea, vomiting, fever, and rash. Laboratory tests revealed ferritin of 42,803 ng/mL, WBC of 20.2 ×10³/μL, CRP of 178 mg/L, and liver enzymes elevated 2–3 fold. It was revealed that he had not been taking tocilizumab for the past 3–4 months. The episode was considered a flare of Still’s disease. Moderate-dose steroids and a three-day course of intravenous anakinra were administered. WES results identified a homozygous *PRF1* c.272C>T (p.Ala91Val) variant, consistent with an HLH susceptibility genotype. WES did not reveal additional pathogenic/likely pathogenic variants in other HLH-associated genes (e.g., UNC13D, STXBP2, STX11, RAB27A, LYST, XIAP), aside from PRF1 A91V. The patient was discharged with maintenance treatment of anakinra (100 mg/day).

**REFERENCES**

[1. Yamaguchi M, Ohta A, Tsunematsu T, Kasukawa R, Mizushima Y, Kashiwagi H, vd. Preliminary criteria for classification of adult Still’s disease. J Rheumatol. Mart 1992;19(3):424-30.](https://www.zotero.org/google-docs/?2EQH1l)

[2. Fautrel B, Zing E, Golmard JL, Le Moel G, Bissery A, Rioux C, vd. Proposal for a new set of classification criteria for adult-onset still disease. Medicine (Baltimore). Mayıs 2002;81(3):194-200.](https://www.zotero.org/google-docs/?2EQH1l)

[3. Henter JI, Horne A, Aricó M, Egeler RM, Filipovich AH, Imashuku S, vd. HLH-2004: Diagnostic and therapeutic guidelines for hemophagocytic lymphohistiocytosis. Pediatr Blood Cancer. 2007;48(2):124-31.](https://www.zotero.org/google-docs/?2EQH1l)

    Supplementary Table 1. Summary of published HLH cases associated with *PRF1* A91V mutation

| **Cases** | **Sex** | **Age at MAS** | **Presentation** | **Treatment** | **Survival Status** | **A91V Homozygous/**  **Heterozygous** | **Other *PRF1* mutation** | **Clinical Findings** | **Haematolo-**  **gical Findings** | **CRP (mg/L)** | **Ferritin (ng/mL)** | **Notable Clinical Features** |
| --- | --- | --- | --- | --- | --- | --- | --- | --- | --- | --- | --- | --- |
| Okur et al [(36)](https://www.zotero.org/google-docs/?sFFN9y)  Case 7 | F | 13 | CNS | HLH-2004 | Alive | Hom | N/A | N/A | N/A | N/A | N/A | 1.5 year survival after diagnosis |
| Okur et al [(36)](https://www.zotero.org/google-docs/?7RAq0J)  Case 8 | M | 12 | Systemic | HLH-2004 | Exitus | Hom | N/A | N/A | N/A | N/A | N/A | 2 months survival after diagnosis |
| Vastert et al [(9)](https://www.zotero.org/google-docs/?uNGlSm)  Case 13 | N/A | N/A | Systemic | N/A | N/A | Het | -499 C>T Ht | Fever, HSM | Thrombocytopenia | 49 mg/L | 82617 | No hemophagocytosis on BMA |
| Vastert et al [(9)](https://www.zotero.org/google-docs/?5XNIW1)  Case 14 | N/A | N/A | Systemic | N/A | N/A | Het | -499 C>T Ht | Fever, HSM | Thrombocytopenia | 300 mg/L | 58265 | Hemophagocytosis on BMA |
| Vastert et al [(9)](https://www.zotero.org/google-docs/?qngmIt)  Case 15 | N/A | N/A | Systemic+CNS | N/A | N/A | Het | -499 C>T Ht | Fever, CNS, HSM | No | 286 mg/L | 46500 | Hemophagocytosis on BMA |
| Zhang et al [(37)](https://www.zotero.org/google-docs/?eglivm)   Patient 3 | F | 18 | Systemic | N/A | N/A | Het | No | N/A | N/A | N/A | N/A | ALL without remission |
| Zhang et al [(37](https://www.zotero.org/google-docs/?amULcA)[)](https://www.zotero.org/google-docs/?broken=1wf4d5)  Patient 4 | F | 18 | Systemic | N/A | N/A | Het | No | N/A | N/A | N/A | N/A | HLH |
| Zhang et al [(37](https://www.zotero.org/google-docs/?kW3Acs))  Patient 5 | M | 18 | Systemic | N/A | N/A | Het | No | N/A | N/A | N/A | N/A | Mycosis Fungoides |
| Zhang et al [(37](https://www.zotero.org/google-docs/?FdGJOO)[)](https://www.zotero.org/google-docs/?broken=irAxvC)  Patient P7 | M | 18 | Systemic | N/A | N/A | Het | 1042 G>A | N/A | Neutropenia | N/A | N/A | Suspected HLH |
| Zhang et al [(37](https://www.zotero.org/google-docs/?VuXtlv))  Patient 8 | M | 19 | Systemic+CNS | N/A | N/A | Het | No | N/A | N/A | N/A | N/A | Vasculitis |
| Zhang et al [(37](https://www.zotero.org/google-docs/?Zlq5l5))  Patient 10 | F | 19 | Systemic | N/A | N/A | Het | 563 C>T | N/A | N/A | N/A | N/A | Suspected HLH |
| Zhang et al [(37](https://www.zotero.org/google-docs/?Xym6H9))  Patient 18 | F | 25 | Systemic | N/A | N/A | Het | No | N/A | N/A | N/A | N/A | Suspected HLH |
| Zhang et al [(37](https://www.zotero.org/google-docs/?Bp13JJ))  Patient 19 | F | 25 | Systemic | N/A | N/A | Het | 445 G>A / 695 G>A | N/A | N/A | N/A | N/A | HLH |
| Zhang et al [(37](https://www.zotero.org/google-docs/?pH9o2Y))  Patient 21 | M | 28 | Systemic | N/A | N/A | Het | No | N/A | N/A | N/A | N/A | Dead sibling because of HLH |
| Zhang et al [(37](https://www.zotero.org/google-docs/?GdjdOg))  Patient 23 | F | 66 | Systemic | N/A | N/A | Het | No | N/A | N/A | N/A | N/A | HLH |
| Zhang et al [(37](https://www.zotero.org/google-docs/?ogCTXw))  Patient 24 | M | 74 | Systemic | N/A | N/A | Het | No | N/A | N/A | N/A | N/A | HLH |
| Zhang et al [(37](https://www.zotero.org/google-docs/?y92XDZ))  Patient 25 | M | 75 | Systemic | N/A | N/A | Hom | No | N/A | N/A | N/A | N/A | HLH |
| Busiello et al [(38)](https://www.zotero.org/google-docs/?Ptd3H9) Proband | F | 13 | Systemic | N/A | N/A | Hom | R231H Ht | Fever,HSM,LAP, | Anemia, Thrombocytopenia, | N/A | Hyperferritinemia(no value) |  |
| Çakan et al [(39)](https://www.zotero.org/google-docs/?9k1kPs)  Case | M | 9 | Systemic | MPZ, ANA, CAN, CsA | Alive | Het | No | Fever, Pericardial effusion, Rash ,Splenomegaly | Anemia, Thrombocytosis,Leucocytosis | N/A | 13670 | ICU-ECMO history |
| Clementi et al [(23)](https://www.zotero.org/google-docs/?P1C8Nd)  Case 1 | M | 27 | Systemic+CNS | MPZ | Alive | Het | Trp374Stop | N/A | N/A | N/A | N/A |  |
| Clementi et al [(23)](https://www.zotero.org/google-docs/?BwXbf0)  Case 2 | F | 25 | Systemic | VIN + CYC + L-Aspa + DOX | Alive | Het | Trp374Stop | N/A | N/A | N/A | N/A | T-cell lymphoblastic lymphoma |
| Sanchez et al [(40)](https://www.zotero.org/google-docs/?lgmkZB)  Patient 1 | F | 0.15 | Systemic+CNS | HSCT | Exitus | Het | No | FEver, cough | Thrombocytopenia, Neutropenia | N/A | 19741 |  |
| Sanchez et al [(40)](https://www.zotero.org/google-docs/?KWJbau)  Patient 2 | M | 5 | Systemic | HLH-2004 + HSCT | Alive | Hom | No | Fever,HSM,Rash,  Dyspnea,Edema | Pancytopenia | N/A | 696 |  |
| Palterer et al [(28)](https://www.zotero.org/google-docs/?hLTnv1)  Case | M | 48 | CNS | MPZ-AZA-IVIG-RTX | Alive | Het | No | Fever,LAP,  Splenomegaly | Pancytopenia | N/A | 726 | Neuromyelitis Optica case |
| Stadermann et al [(24)](https://www.zotero.org/google-docs/?KTWFcb)  Case | F | 20 | Systemic | HLH-2004 | Exitus | Het | R104C | Fever | Pancytopenia | N/A | 1032 |  |
| Ueda et al [(25)](https://www.zotero.org/google-docs/?Wed1zH)  Case | N/A | 3.3 | Systemic+CNS | N/A | N/A | Het | R54C, 285delK | N/A | N/A | N/A | N/A |  |
| Gomez et al [(41)](https://www.zotero.org/google-docs/?evzBq9)  Case | F | 22 | Systemic | MPZ + CsA | Alive | Het | No | Fever,Rash,LAP | Leucopenia, Thrombocytopenia | N/A | 9445 | EBV infection |
| Girschick et al [(42)](https://www.zotero.org/google-docs/?nANWZo) Case | F | 16 | Systemic | MPZ+CsA | Alive | Het | No | Fever, Splenic infarct, Renal failure,Cardiac failure | Anemia, thrombocytopenia | N/A | N/A |  |
| Mancebo et al [(43)](https://www.zotero.org/google-docs/?fGxGa0) Case | M | 49 | Systemic | HLH-94+Antibiotics | Alive | Hom | No | Periorbital Edema | Pancytopenia | N/A | 7000 | Tuberculosis sacroileitis |
| Jang et al [(44)](https://www.zotero.org/google-docs/?GxzVTi)  Case | M | 21 | Systemic | IVIG-RTX-MPZ | Alive | Hom | No | Fever,LAP, HSM, Pericardial effusion,Pulmonary involvement | Anemia, thrombocytopenia | N/A | 17846 | EBV infection |
| Al-Samkari et al [(45)](https://www.zotero.org/google-docs/?5jfnny) Case | F | 58 | Systemic | HLH-94+Antibiotics | Alive | Het | No | Fever, Rash,Renal failure,Cardiac failure | Anemia, thrombocytopenia | N/A | 32640 | Breast cancer on pembrolizumab treatment |
| Schulert et al [(46)](https://www.zotero.org/google-docs/?jwa6Jm) Patient 7 | N/A | N/A | Systemic | N/A | Exitus | Het | No | fever | Thrombocytopenia | N/A | >3000 | H1N1 mortality |
| Schulert et al [(46)](https://www.zotero.org/google-docs/?4gwUIX) Patient 11 | N/A | N/A | Systemic | N/A | Exitus | Het | No | Fever, Splenomegaly, LAP | Anemia, thrombocytopenia | N/A | N/A | H1N1 mortality |
| Mehda et al [(47)](https://www.zotero.org/google-docs/?Crzdwf) Case | M | 22 | Systemic | MPZ, Etoposide, RTX, TCZ, HSCT | Alive | Het | No | Fever, Splenomegaly | Pancytopenia | N/A | >40000 | EBV infection , Sezary after transplantation |
| Cabrera-Marante et al [(48)](https://www.zotero.org/google-docs/?LQylrr) Patient 1 | F | 45 | Systemic + CNS | MPZ, Antibiotics,IFN beta | Exitus | Het | No | Confusion, Dyspnea | Leucocytosis | 22.9 | 1107 | COVID-19 infection |
| Cabrera-Marante et al [(48)](https://www.zotero.org/google-docs/?bLVyGp) Patient 2 | M | 46 | Systemic | MPZ, TCZ | Exitus | Het | No | Fever, Cough | None | 23.9 | 3032 | COVID-19 infection |
| Aviner et al [(49)](https://www.zotero.org/google-docs/?Jw6lCV) case | M | 0.1 | Systemic | DXM | Alive | Het | No | Fever, Rash | Thrombocytopenia | N/A | 4165 | Human Parechovirus Infectioon |
| Bailo et al [(21)](https://www.zotero.org/google-docs/?rDOZ33) case | F | 72 | Systemic + CNS | HLH-2004 , Antibiotics | Exitus | Het | No | Fever,Hepatosplenomegaly, Rash | Pancytopenia | 66.5 | 9193 | Past bladder cancer |
| Our Case | M | 19 | Systemic | IL1 , IL6 , MTX , MPZ-DXM | Alive | Hom | No | Fever, Hepatosplenomegaly , LAP , Rash , Arthritis | Anemia, Thrombocytopenia | 291 | 91391 |  |

Table 1. (continued)

Supplementary Table 2. Univariate and Stepwise Multivariate Logistic Regression Analyses of Clinical and Laboratory Parameters Predicting A91V Variant HLH Cases

|  | Univariate | Multivariate | | | | |
| --- | --- | --- | --- | --- | --- | --- |
| Parameter | OR | Step 1 | Step 2 | Step 3 | Step 4 | Final Model |
| Age >30 years | 0.36 (0.13 - 096; p=0.04) | 0.14 (0.01 – 1.95; p=0.14) | 0.14 (0.01 – 1.96; p=0.14) | 0.20 (0.02 – 2.03; p=0.17) |  |  |
| Fever | 0.09 (0.008 - 1.19; p=0.07) | 0.53 (0 – 80619.3; p=0.91) |  |  |  |  |
| Splenomegaly | 2.41 (0.55 - 10.42; p=0.23) | 2.40 (0.16 – 34.45; p=0.51) | 2.44 (0.17 – 34.39; p=0.50) |  |  |  |
| Lympadenopathy | 0.29 (0.06 - 1.39; p=0.12) | 0.10 (0.007 – 1.37; p=0.08) | 0.1 (0.007 – 1.34; p=0.08) | 0.13 (0.012 – 1.38; p=0.09) | 0.19 (0.02 – 1.70; p=0.13) | 0.16 (0.02 - 1.32; p=0.09) |
| Rash | 0.21 (0.04 - 1.02; p=0.05) | 0.08 (0.007 – 1.08; p=0.05) | 0.08 (0.007 – 1.07; p=0.05) | 0.06 (0.006 – 0.82; p=0.03) | 0.08 (0.008 – 0.90; p=0.04) | 0.14 (0.01 - 1.13; p=0.06) |
| Serosal Effusion | 1.88 (0.45 - 7.86; p=0.38) | 0.11 (0.008 – 1.64; p=0.11) | 0.11 (0.008 – 1.59; p=0.1) | 0.14 (0.01 – 1.83; p=0.13) | 0.18 (0.01 – 2.15; p=0.17) |  |
| Ferritin >7.000 | 7.0 (1.67 - 29.22; p=0.008) | 41.44 (2.78 – 617.4; p=0.007) | 41.81 (2.82 – 619.5; p=0.007) | 37.78 (2.99 – 476.3; p=0.005) | 29.37 (2.71 – 317.7; p=0.005) | 17.3 (2.0 - 146.3; p=0.009) |
